# Editorial: HPV natural history, immunological responses and vaccination strategies: challenges and opportunities

**DOI:** 10.3389/fimmu.2023.1349664

**Published:** 2024-01-08

**Authors:** Zheng Quan Toh, Fanghui Zhao, Lei Zhang, Lanlan Wei, Huachun Zou

**Affiliations:** ^1^ Department of Paediatrics, The University of Melbourne, Parkville, VIC, Australia; ^2^ Infection, Immunity and Global Health, Murdoch Children’s Research Institute, Parkville, VIC, Australia; ^3^ Department of Cancer Epidemiology, National Cancer Center/National Clinical Research Center for Cancer/Cancer Hospital, Chinese Academy of Medical Sciences and Peking Union Medical College, Beijing, China; ^4^ China-Australia Joint Research Center for Infectious Diseases, School of Public Health, Xi’an Jiaotong University Health Science Center, Xi’an, Shaanxi, China; ^5^ Melbourne Sexual Health Centre, Alfred Health, Melbourne, VIC, Australia; ^6^ Central Clinical School, Faculty of Medicine, Nursing and Health Sciences, Monash University, Melbourne, VIC, Australia; ^7^ National Clinical Research Center for Infectious Diseases, Institute for Hepatology, The Third People’s Hospital of Shenzhen, Guangdong, China; ^8^ Southern University of Science and Technology, Shenzhen, Guangdong, China; ^9^ School of Public Health, Fudan University, Shanghai, China; ^10^ Fujian Maternity and Child Health Hospital, College of Clinical Medicine for Obstetrics and Gynecology and Pediatrics, Fujian Medical University, Fuzhou, Fujian, China; ^11^ School of Public Health, Southwest Medical University, Luzhou, China; ^12^ Kirby Institute, University of New South Wales, Sydney, Australia

**Keywords:** human papillomavirus (HPV), vaccination, cervical cancer, women, men who have sex with men (MSM), tumor microenvironment, cost-effectiveness, knowledge and attitude

Human papillomavirus (HPV) infection is the most common sexually transmitted infection, with up to 90% of individuals infected with HPV at some point in their lives (1). While most individuals can clear the infections without any interventions within 1-2 years (2), persistent infection can cause a range of diseases from benign lesions, such as genital warts, to cancer of the anogenital regions, in particular cervical cancer and also cancers in the head and neck region (2).

The link between HPV and cervical cancer was first reported in the 1980s (3). It then took around 20 years of research before highly effective HPV vaccines were available to prevent HPV infection (4). However, HPV vaccines remain inaccessible to many low- and middle-income countries as a result of vaccine inequity. There has been significant progress in HPV research in the past two decades, including improved understanding of HPV infection and natural HPV immunity, new vaccine schedules as well as more effective diagnostic and treatment strategies to prevent cervical cancer and other HPV-associated diseases (5, 6). The improved knowledge and strategies have led to the adoption of the global strategy (90% of girls fully vaccinated with the HPV vaccine by the age of 15; 70% of women screened using a high-performance test by the age of 35, and by the age of 45; 90% of women with pre-cancer treated and 90% of women with invasive cancer managed) to eliminate cervical cancer in the coming century (4).

This collection of articles under this Research Topic covers a range of the latest research on HPV including HPV and host immune responses, HPV vaccines, HPV infection in men who have sex with men (MSM), as well as implementation research on knowledge of HPV infection, and attitudes towards HPV vaccination and screening.

In this Research Topic, Hewavisenti et al. provided a comprehensive overview of HPV and cervical cancer, with an emphasis on HPV infection in the context of immunodeficiency. Immunocompromised individuals, including those infected with human immunodeficiency virus, are at a particularly high risk of developing HPV-associated cancers. In addition, the review article by Wu et al. highlighted the important management strategy for cervical cancer screening in the current era of post-HPV vaccination, including the use of highly sensitive HPV DNA testing.

The local cellular microenvironment plays a crucial role in the HPV carcinogenesis. In this Research Topic, Dai et al. discussed the interaction between HPV and Langerhans cells, an important subset of antigen-presenting cells in the cervical epithelium that is responsible for eliciting cellular immunity and eliminating HPV infection. The authors highlighted how cervicovaginal microbiome can influence HPV persistence and proposed research strategies targeting Langerhan cells to facilitate HPV clearance. In addition, three original research articles in this Research Topic investigated the immune cells involved in cervical and head and neck carcinogenesis. The first paper by Zhang et al. applied sophisticated computational methods to characterise the infiltration patterns of the microenvironment during cervical cancer progression. They identified 31 immune cells, which can be grouped into 5 clusters of cells based on their numbers during disease progression. The second paper by Song et al. identified two immune subtypes of HPV positive cervical cancers that differs in their immune cells’ composition, stromal contents, DNA repair activity, proliferation and overall survival prognosis. This improves our understanding of tumour immunity in cervical cancer and advance immunotherapy research on cervical cancer. The third paper by Jiang et al. identified a unique macrophage subset with T cell receptor and CD3-specific signatures in HPV-positive head and neck cancers (HNC), improving our understanding of the immune landscape of HPV-related HNC. Overall, these studies inform future studies about investigating novel therapies for HPV viral clearance and/or lesion regression through targeting the immune cells at the microenvironment.

The prophylactic HPV vaccines are highly immunogenic and effective in preventing HPV infection and cervical cancer (7). However, the high vaccine cost has impeded the global use of HPV vaccine, particularly in low- and middle-income countries where the burden of cervical cancer is the highest (8). In this Research Topic, Li et al. summarised the evidence of the immunogenicity, efficacy and safety of five currently licensed HPV vaccines in China, including two recent China-made HPV vaccines, which are thought to be cheaper to produce. These new vaccines, as well as a quadrivalent HPV vaccine from serum institute of India will alleviate the global HPV vaccine supplies and will have a significant impact on improving HPV vaccine access, particularly in low- and middle- income countries.

Infection with HPV affects both males and females. While studies have shown that female-only HPV vaccination program offers direct protection to females and also herd protection to males (9), populations such as men who have sex with men (MSM) do not have the herd protection from female-only HPV vaccination program and remain at risk of HPV-associated diseases, including penile and anal cancers. Three research papers in this Research Topic documented the HPV prevalence in MSM. Zhang et al. reported high incidence of anal HPV infection (53.8 per 1000 person-month), with more than a third of high-risk HPV types in a cohort of HIV-negative MSM based in Xinjiang, China. The most common HPV types were HPV16 and HPV6 and the clearance rate for these types were the lowest. In the same cohort, Liu et al. found that around 30% were reinfected with the HPV types contained in the nonavalent HPV vaccine. Zhou et al. also reported a similarly high anal HPV incidence rate (43.6 per 1000 person-month) and penile HPV infection (26.8 per 1000 person-month) in MSM based in Taiwan. Taken together, these studies indicate a high prevalence of HPV infection in MSM and vaccinating MSM regardless of prior exposure, will protect this high-risk group against HPV-associated diseases.

This Research Topic also included several research articles on the knowledge, attitudes and perspective of HPV infection and HPV vaccination in different populations. Domínguez-Riscart et al. conducted a cross-sectional study in Spain to evaluate knowledge about HPV-related diseases among caregivers of trans-adolescent girls. The authors found more than three-quarters of the caregivers had poor knowledge of HPV infection and that adolescent trans girls had low intention of vaccination against HPV. A cross-sectional study by Paduano et al. based in Italy reported only around one-third of adult women were aware that oropharyngeal cancer can be caused by HPV. These two studies indicate the urgent need to increase the awareness of HPV-related diseases and the prevention strategies available, particularly among groups that are at risk of HPV infection. Chan et al. reported a preference for HPV vaccination instead of HPV screening among MSM in Hong Kong, suggesting that HPV disease prevention in this group should be focused on HPV vaccination. However, the high vaccine cost is a major obstacle against the scale-up of HPV vaccination in MSM in China, as highlighted by Li et al. Further modelling based on the new HPV vaccines from China and India, as well as the reduction in doses, would inform vaccine strategies among MSM in China as well as globally.

This Research Topic covers a range of HPV Research Topics that inform further research in designing innovative immunotherapeutics to clear HPV infection and regress lesions, as well as implementation research to improve vaccine accessibility for both the general population and high-risk groups such as MSM, which ultimately contributes to the reduction of HPV-associated diseases, as well as the acceleration of cervical cancer elimination strategy.

## Author contributions

ZT: Conceptualization, Writing – original draft, Writing – review & editing. FZ: Writing – review & editing. LZ: Writing – review & editing. LW: Writing – review & editing. HZ: Writing – review & editing.
